# New e-health services for the European Network for Rare and Congenital Anaemias (e-ENERCA)

**DOI:** 10.1186/1750-1172-9-S1-P9

**Published:** 2014-11-11

**Authors:** J L Vives Corrons, M Mañú Pereira, L Olaya

**Affiliations:** 1 Red Cell Pathology Unit, Hospital Clínic. University of Barcelona, Spain

## 

Institut d'Investigacions Biomèdiques August Pi i Sunyer (IDIBAPS), Barcelona, Spain

Rare Anaemias (RA) are a group of Rare Diseases (RD) with prevalence, in Europe, less than 5 per 10.000 individuals. Major forms of RAs require red blood cell transfusions, iron chelation, splenectomy, and/or in very severe cases, bone marrow transplantation, as main therapeutic options. Beta-thalassaemia major is predominant in Italy and Cyprus, and sickle cell disease (SCD) in African population. During the last 30 years, SCD is increasing in Europe due to African immigration, leading to an important impact on health care burden in several countries. Preventive programs, aiming to epidemiological control, and improvement of diagnosis and clinical management of major RA, are crucial for decreasing the affected birth rate and achieving an efficient balance between morbidity and patient’s life expectancy. Since 2003, the European Network for Rare and Congenital Anaemias (ENERCA) has taken an active role for improving this situation by the following actions: a) the identification of Centres of Expertise on RAs in Europe according to the recommendations of ENERCA White Book b) the promotion of best clinical and laboratory practices by the publication of ENERCA recommendations c) the improving of continuous medical education, by organising topic-specific training courses, workshops and symposia, e) the empowerment of patients, by cooperation with Patient’s Associations, and co-organizing a bi-annual European Symposium on RAs with interactive patients-health professionals sessions. In September 2013, a new phase of the project called e-ENERCA has started with the aim to provide, patients and professionals with e-Health tools for assure the same access to health services in RAs across Europe, independently from the country of practise and origin of the patients. e-Health services will be developed through the set-up of three different e-platforms endorsed by ENERCA website (http://www.enerca.org) : 1) **e-Registry**, a Pan European registry of RAs for gathering patient’s data necessary to achieve the required sample size for epidemiological surveillance and clinical research 2) **e-Learning** , a teaching platform for the dissemination of knowledge, continuous medical education, and best practices awareness and promotion through Internet, and 3) **e-Medicine** , a platform to provide, at distance, expertise (telexpertise) and diagnostic facilities (telediagnosis), avoiding, when possible, the need of travelling. Finally, e-ENERCA will also promote the recognition of the previously identified Centres of Expertise in RAs (White Book) by the national health authorities, a mandatory condition for ENERCA final recognition as *European Reference Network in Rare Anaemias* (*RA-ERN*).

